# Spatiotemporally Detailed Quantification of Air Quality Benefits of Emissions Reductions–Part I: Benefit-per-Ton Estimates for Canada and the U.S.

**DOI:** 10.1021/acsestair.4c00127

**Published:** 2024-09-03

**Authors:** Shunliu Zhao, Petros Vasilakos, Anas Alhusban, Yasar Burak Oztaner, Alan Krupnick, Howard Chang, Armistead Russell, Amir Hakami

**Affiliations:** † Department of Civil and Environmental Engineering, Carleton University, Ottawa, Ontario K1S 5B6, Canada; ‡ School of Civil and Environmental Engineering, Georgia Institute of Technology, Atlanta, Georgia 30331, United States; § Resources For the Future, Washington, D.C. 20036, United States; ∥ Emory University, Atlanta, Georgia 30322, United States

**Keywords:** Air quality, emission reduction, particular matter, health benefit, benefit per ton, CMAQ adjoint

## Abstract

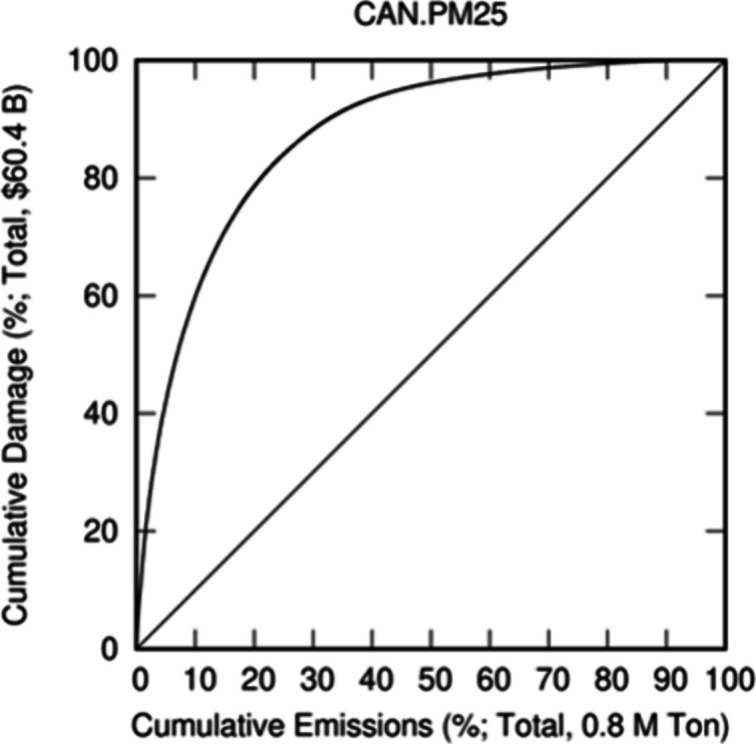

The U.S. EPA’s Community Multiscale Air Quality (CMAQ)-adjoint model is used to map monetized health benefits (defined here as benefits of reduced mortality from chronic PM_2.5_ exposure) in the form of benefits per ton (of emissions reduced) for the U.S. and Canada for NOx, SO_2_, ammonia, and primary PM_2.5_ emissions. The adjoint model provides benefits per ton (BPTs) that are location-specific and applicable to various sectors. BPTs show significant variability across locations, such that only 20% of primary PM_2.5_ emissions in each country makes up more than half of its burden. The greatest benefits in terms of BPTs are for primary PM_2.5_ reductions, followed by ammonia. Seasonal differences in benefits vary by pollutant: while PM_2.5_ benefits remain high across seasons, BPTs for reducing ammonia are much higher in the winter due to the increased ammonium nitrate formation efficiency. Based on our location-specific BPTs, we estimate a total of 91,000 U.S. premature mortalities attributable to natural and anthropogenic emissions.

## Abstract 2

Due to the spatiotemporal variabilities in benefit per ton of emission reductions, reducing 20% of the primary emissions would result in over half the societal health benefits in both the U.S. and Canada.

## 1 Introduction

Chemical transport models (CTMs) are commonly used to evaluate emissions control options and produce information for policy makers to formulate strategies for improving air quality.^1,2^ One metric that succinctly provides useful information to help identify effective policies is the monetized societal benefit associated with reducing 1 t of emissions of a specific pollutant, commonly known as benefit per ton (BPT) value (or marginal benefit (MB) in environmental economics).^3,4^ BPTs are primarily driven by the degree to which reducing emissions reduces the risk of premature mortality,^2,5−8^ directly relating pollutant source emissions to human health. BPTs can also include potential benefits for other health and environmental outcomes.

One of the most commonly used method to calculate source impact estimates such as BPTs is to model a given case twice, once as a baseline and afterward with an incremental perturbation to the emission source of interest, with the difference between the two cases serving as the impact of the emission source(s) on health or environmental outcome. This is known as the brute-force method, and while it has the advantage of simple implementation and limited computational resource requirements, it becomes increasingly burdensome for cases where multiple sources are concerned. For such cases, two approaches are used. In one, either pollution sources are grouped together across time and based on type and sector,^3^ region, or both.^4^ This grouping drastically reduces the number of sources studied for the calculation of BPTs; such methods however cannot distinguish between the health impact of emissions at different locations or times. The second approach uses reduced form models (RFMs), which are simplified (reduced) representations of a CTM to estimate source impacts in fashions that are computationally less intensive and allow for BPT estimation for multiple sources.^9−13^ Due to the simplified nature of an RFM however, the robustness and accuracy of the results can be compromised.^14,15^

Here we use adjoint modeling as a full complexity method to calculate BPTs for the U.S. and Canada. Instead of lumping sources and emission species or using simplifications, the adjoint method is a full-form model and simulates air quality impacts for all sources and pollutants of interest across the entire domain at all times in a way that includes information on how the benefits vary based on the specific location and time of emissions. This method is sometimes referred to as reverse or receptor-oriented modeling because it tracks the impact on the desired location(s) (receptors) backward in time to the emission locations, instead of a forward method that follows the emissions from the source to the point of impact. We use the adjoint method to investigate reduced mortality outcomes and monetized benefits from chronic exposure to PM_2.5_, and to create a database of location-specific BPT estimates for Canada and the U.S., as a measure of the societal benefits associated with reducing emissions from transportation and other select sources.

## 2 Materials and Methods

The main tool used in this study is the adjoint-enabled version of the U.S. EPA’s Community Multiscale Air Quality (CMAQ) model, one of the most widely used CTMs globally. CMAQ is a state-of-the-art and comprehensive photochemical CTM that accounts for the transport and transformation of gas, particle, and aqueous-phase pollutants in the atmosphere.^16^ Like the underlying CTM, the adjoint model follows the atmospheric transformation and transport of an emitted pollutant (e.g., NOx) to its impact on other species (e.g., ozone or particulate matter), but in reverse order.

CTMs are considered source-based models as they are designed to simulate the transport and transformation of pollutants from the point of release to the atmosphere (i.e., source) to the point of impact (receptor). The adjoint model is designed to do the opposite; it starts at the receptors and traces impacts back in time and through all the atmospheric processes to the point of origin, i.e., the specific location of the source. To do so, the adjoint model “advances” backward in time, i.e., an adjoint simulation starts at the end and ends at the beginning. As the model marches backward in time, it solves and integrates a system of equations that are distinct but related to the governing equations of the underlying CTM (in this case, CMAQ).

### 2.1 Adjoint Model

An adjoint version of CMAQ v5.0 was developed as described in detail elsewhere.^17−19^ The adjoint version of CMAQ, referred to as CMAQ-ADJ hereafter, has undergone extensive testing and evaluation and has been shown to produce estimates consistent with the original CMAQ model.^20^

To apply the adjoint method to a policy-relevant problem such as health impact assessment, two conditions need to be met. First, it should be possible to condense the policy question for which source impacts are sought into a single metric, referred to as the adjoint cost function. Multiple cost functions can be treated by multiple applications of CMAQ-ADJ. For example, and as is the case for this study, the policy question can be the impact of air quality on population health, as represented by premature deaths due to long-term exposure to PM_2.5_. In this study, this policy question is reduced to a scalar metric, i.e., the total counts or monetary valuation of premature deaths attributed to PM_2.5_ exposure in Canada or the U.S. Note that in our example, generating estimates for Canada and the U.S., separately, constitutes two different adjoint cost functions, requiring two sets of adjoint simulations. The second condition requires that the relationship between the adjoint cost function and concentrations of pollutants are known quantitatively. In this case, the relationship between premature mortality (and its valuation) is described by concentration response functions (CRFs), derived from epidemiological models and valuation estimates for each country.

### 2.2 Episode Selection

During the backward simulation, CMAQ-ADJ requires concentrations of all pollutants at all times. Therefore, the adjoint/backward simulation has to be preceded by a forward simulation of the original CMAQ model during which all concentrations are saved (or checkpointed) for use in the CMAQ-ADJ model. This requires significant storage capacity. This storage requirement rapidly becomes large at high model resolutions which renders long simulation periods (e.g., yearlong) at fine resolutions computationally infeasible. For this reason, adjoint simulations at finer resolutions are often conducted for shorter periods than regular CMAQ simulations to address this computational limitation.

Our base BPT simulations are conducted for the contiguous U.S. and the more populous parts of Canada (covering 97.3% of the Canadian population) at a 12 km horizontal resolution. For this study we use 2-week episodes to represent BPT estimates for each season. In other words, annual estimates of source impacts are constructed from four seasonal estimates, each of which is based on the simulation of a 2-week representative period.

The episodes are selected by identifying four 2-week periods that best represent the season, and were independently identified for Canada and the U.S. We choose seasonal episodes based on an anomaly analysis of the entire year (by season). To conduct our anomaly analysis, we generate adjoint-based BPTs for the entire year at a coarser resolution (36 km) where yearlong simulations are possible. We then form normalized bias functions defined as the domain-wide bias for 2-week representation of the season for each possible 2-week period in the season (Table A1). Episodes are selected for each country separately, but the same episode is applied to all regions in the country. Please refer to Appendix A for details of episode selection and list of episodes. The errors associated with episode selection are assessed in detail and reported elsewhere.^21^

### 2.3 CMAQ and CMAQ-ADJ Inputs and Simulations

Conducting adjoint simulations relies on similar model inputs as a regular CTM simulation. For CMAQ and CMAQ-ADJ simulations, these inputs include gridded emissions, meteorological parameters (e.g., wind, temperature, precipitation, cloudiness, etc.), and pollutant concentrations for model initialization and at the lateral boundaries (initial and boundary conditions).

Emission inventories used in this study are derived from the 2016 Emission Inventory Platform (beta version) prepared by the U.S. National Emission Inventory Collaborative (NEIC). NEIC is a partnership between the U.S. EPA, other federal agencies, and various state agencies responsible for air quality management in the U.S. The partnership was established to provide consistent, reliable, and accessible data for photochemical air quality modeling in the U.S. In this study we use the beta version as that was the available version at the time of our study implementation period. 2016 platform emissions are derived from the 2014 version of the National Emission Inventory (NEI) by applying adjustments to various emission sectors.^22^ These modifications include any additional state and local information that was available for the year 2016.

The platform provides emissions for the CB6 chemical mechanism with aerosol version AE7; however, the CMAQ-ADJ uses the CB5 chemical mechanism and AE5 aerosol species. Emissions were mapped accordingly to the alternative mechanisms for gas-phase and aerosol species. The platform provides emissions at 12 and 36 km horizontal resolution, but also includes the necessary data and information to generate emissions at 4 km resolution. CMAQ simulations also require boundary conditions or concentrations at the lateral boundaries of the domain for the simulation period. Boundary conditions are provided from simulations by the Hemispheric version of CMAQ (H-CMAQ). H-CMAQ was applied to the northern hemisphere using a 108 km resolution using the 2016 Global Hemispheric Emission Inventory Platform^23^ incorporating regional updates and improvements (particularly over North America and China) to the Hemispheric Transport of Air Pollution version 2 (HTAPv2) emission inventory. Meteorological data are also available in 2016 v7.2 platform and were prepared using the Weather Research and Forecasting model (WRF) version 3.8^24^ at 12 and 36 km resolutions with 35 vertical layers.

CMAQ simulations are conducted over a continental domain that covers the contiguous U.S. and most of Canada, capturing the overwhelming majority (97.3%) of the Canadian population. The 12-km computational domain has 35 vertical layers that extend well into the stratosphere. The vertical structure of the model is nonuniform and has a higher resolution (i.e., shallower layer depths) closer to the surface where emissions and impacts are more significant than aloft. Forward 12 km simulations have normalized mean bias (NMB) and normalized mean error (NME) of 3% and 27%, respectively, which are well within recommended performance criteria.^25^

For the 36 km domain, annual adjoint simulations are performed to provide a basis for episode selection as described above. Episodic 12 km simulations have ramp-up periods (i.e., added days) to ensure that the simulation period starts with representative concentrations and is free from the impact of initialization. Ramp-up periods apply to both forward and adjoint runs; adjoint simulations start for a period after the end of the episode. For the 36 km annual simulations, 10-day and 4-day ramp up periods are used for forward and adjoint simulations, respectively. For 12 km simulations shorter ramp-up periods (1 day for both forward and adjoint simulations) are used, as both forward and adjoint simulations start with interpolated 36 km (or 12 km for 4 km domains) concentrations which have already included ramp-up periods.

### 2.4 Epidemiological Model

Over the last 15 years, a number of studies have provided effect estimates for chronic PM_2.5_ exposure in Canada, the U.S., and globally using various cohorts.^26−34^ We use the Global Exposure Mortality Model (GEMM)^27^ as our primary epidemiological model for our BPT estimates. GEMM is a pooled cohort informed by 41 individual cohorts from 16 countries and covers the range of concentrations seen across the globe. The choice of GEMM allows for the use of a single model in both Canada and the U.S. GEMM predicts globally higher estimates of air pollution burden than the Global Burden of Disease (GBD) studies, but North American estimates that are more in line with previous studies. For BPT estimations we use GEMM effect estimates for noncommunicable diseases and lower respiratory infections (NCD+LRI) with population and baseline rates for adults 25 years and older. GEMM has a sublinear (concave) CRF, with increasing rate of reduction in hazard ratio (HR) at lower concentrations. While we present BPTs for GEMM CRF in this study, a sensitivity analysis of BPT estimates based on 5 other CRFs^30,32,34,35^ can be found elsewhere.^21^

GEMM is formulated into the adjoint cost function based on the following equations:^27^
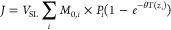
1where
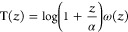
2
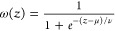
3
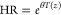
4and
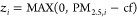
5*M*_0, *i*_ and *P*_*i*_ are baseline mortality rates and population (age >25) at location *i*, respectively, *cf* indicates counterfactual concentration, and *HR* is the hazard ratio from the model. The coefficients in the above equations have the following values:^27^ θ = 0.1231, α = 1.5, μ = 10.4, ν = 25.9, and *cf* = 2.4 μg m^–3^. Population data is generated using respective census information in Canada and the U.S. and is mapped to various grid resolutions. Similarly, baseline rates are taken from USEPA’s Benefit Mapping and Analysis Program (BenMAP)^36^ and Health Canada’s Air Quality Benefit Assessment Tool (AQBAT)^37^ for U.S. and Canada, respectively, and are mapped/aggregated onto appropriate horizontal resolution in various simulations.

### 2.5 Valuation

We use a U.S. and Canadian Value of Statistical Life (VSL) for monetizing premature mortality counts, based on EPA and Health Canada practices in benefit-cost analyses of proposed regulations as embodied in the BenMAP model in the U.S. and AQBAT in Canada. In addition to being the standard valuation method of choice for the American and Canadian governments, the VSL, either derived from surveys (termed stated preference studies) or from real world observations (termed revealed preference studies) is the recommended concept for valuing reductions in mortality risks from regulations.^38−42^

We use VSLs of $10.2 M (2016 USD, with income adjustment) for the U.S.^41^ and $7.5 M (2016 CAD) for Canada.^38^ We follow the U.S. EPA’s recommended approach to apply a cessation lag between the timing of reduction in PM_2.5_ exposure and the realization of mortality reductions. We will apply the recommended 20-year distributed lag model with 30% of deaths occurring in the first year, 50% in years 2–5, and the remaining 20% in years 6–20 for PM_2.5_,^43^ and a social discount rate of 3% per year.^40^ This results in an overall discounting factor of 0.90606 for the U.S.^15^ For Canada, a cessation lag is not officially recommended and therefore is not applied. As mentioned before, our valuation is solely based on the VSL and does not account for morbidity, disability, and loss of quality of life.

## 3 Results

**Figure 1:**
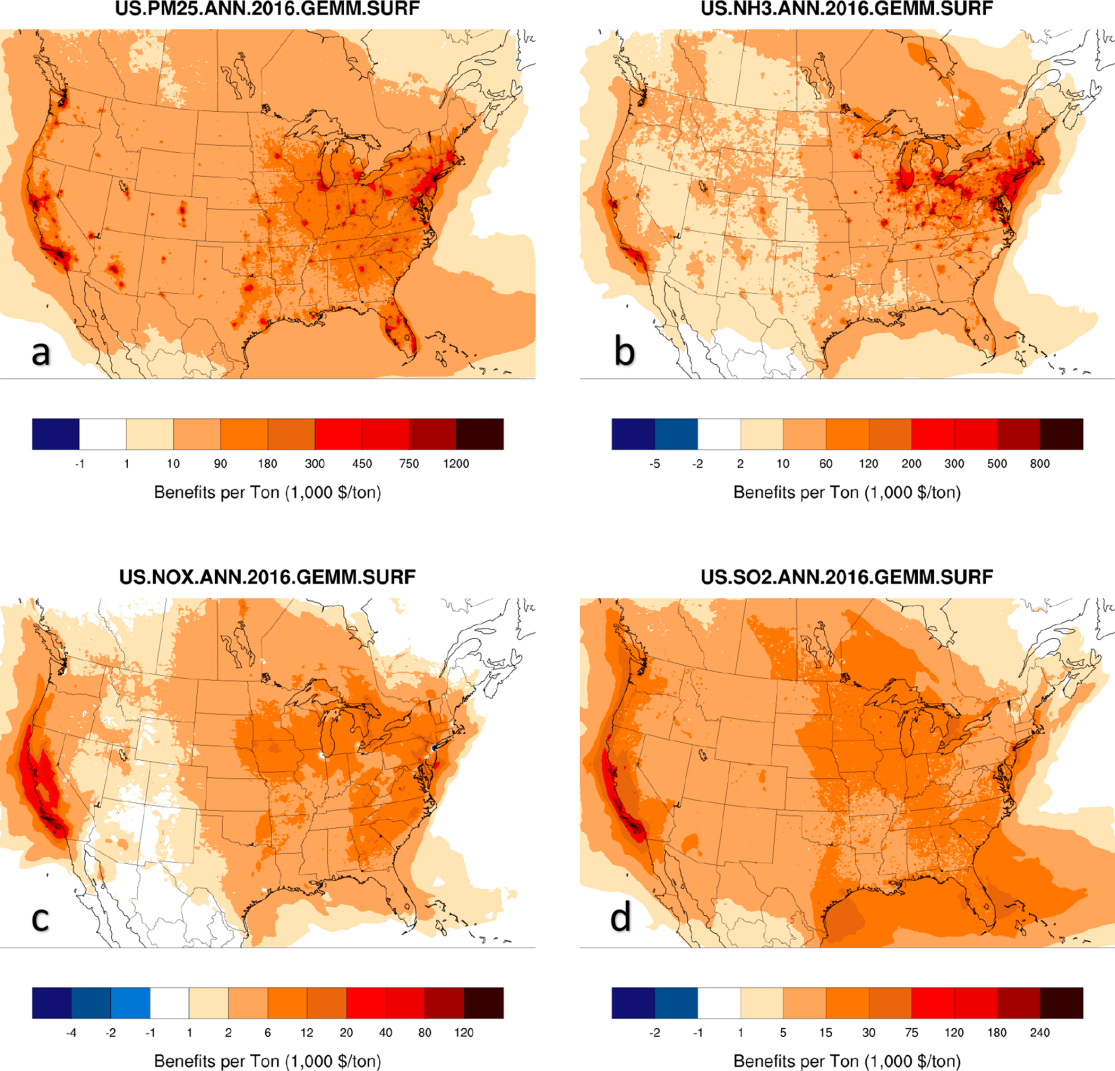
Surface (annual) U.S. BPTs for various species [(a) PM_2.5_, (b) NH_3_, (c) NO_*x*_, and (d) SO_2_] and the GEMM CRF. Individual plot titles indicate the country and species for which the plot is generated. U.S. BPT estimates are for 2016 USD. All the results shown are BPTs for surface sources. For each species, a value on a location in the map indicates the monetized societal benefits of reducing emissions of that species by 1 metric ton. For example, a value of $1M in New York City for PM_2.5_ BPT implies that reducing primary PM_2.5_ emissions at that location in the city (grid cell) leads to a $1M population health benefit in the form of avoided mortality across the entire country.

**Figure 2:**
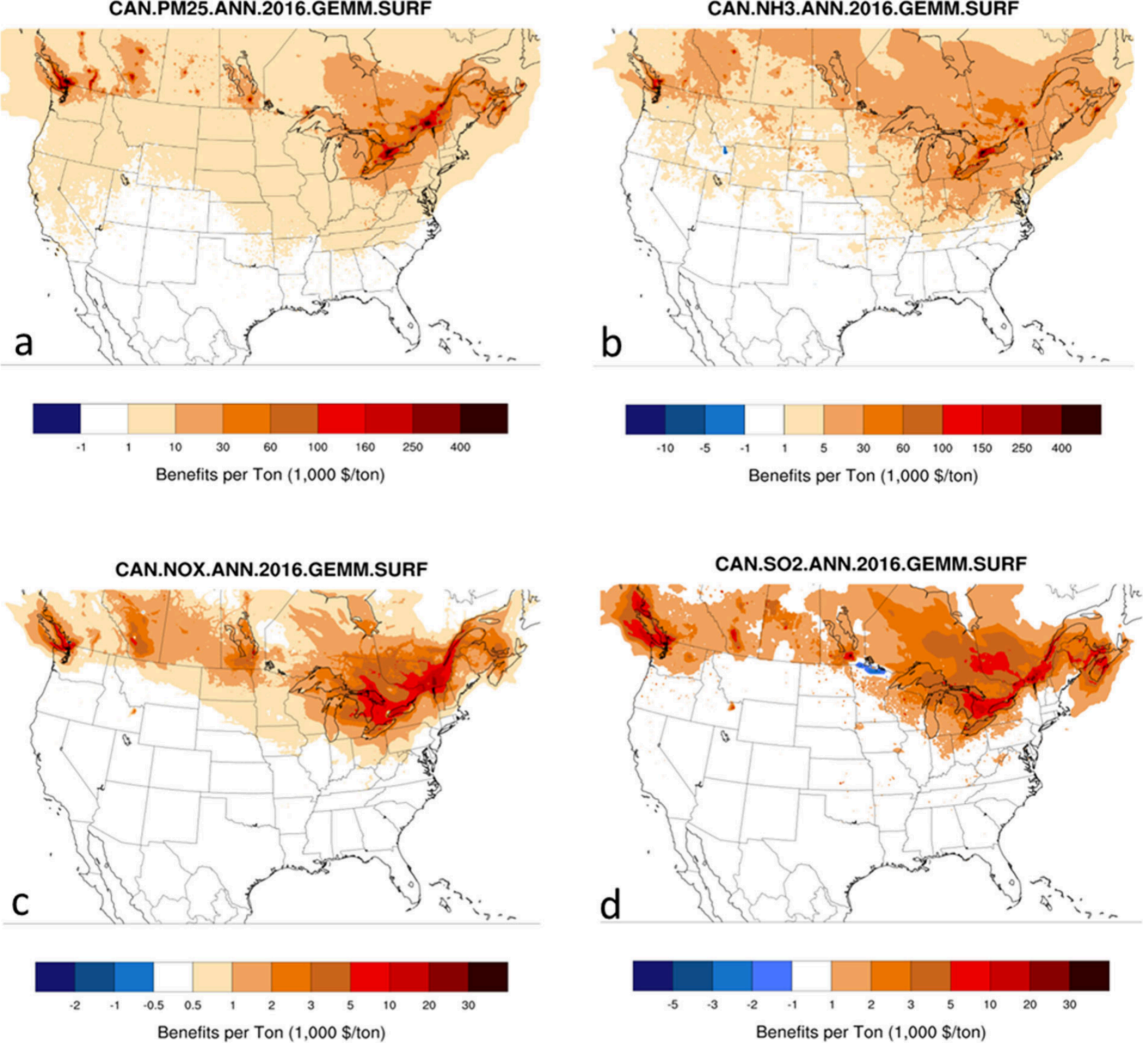
Surface (annual) Canadian BPTs for various species [(a) PM_2.5_, (b) NH_3_, (c) NO_*x*_, and (d) SO_2_)] and the GEMM CRF. Individual plot titles indicate the country and species for which the plot is generated. Canadian BPT estimates are for 2016 CAD.

Location-specific BPTs for the U.S. and Canada show a large variation in valuation (Figures 1, 2). Note that while adjoint-based BPTs are location-specific and provide granular information about source impacts at all locations, they do not contain any information about the location of impact as the benefits are integrated across the domain. In other words, the adjoint BPTs provide source location specificity but at the expense of receptor location specificity. Particulate matter and ammonia emissions (Figure 1a,b) have the greatest per-tonne of emissions benefits, followed by SO_2_ (Figure 1d) and NO_*x*_ (Figure 1c). Proximity to population centers is a main driver for BPTs and as such, California and parts of the Northeastern U.S. (Pennsylvania, New York, etc.) tend to have the highest BPT values. While for primary PM_2.5_ emissions, population distribution is the main driver of BPTs, for secondary inorganic PM_2.5_ precursors, chemical regime and conditions conducive to gas-to-particle conversion become equally important. For example, SO_2_ shows larger BPTs in coastal areas with downwind populations where aqueous phase oxidation to sulfate is facilitated. Similarly, in areas of high NOx (e.g., urban areas), ammonia has higher efficiency in producing ammonium nitrate and shows large BPTs, while higher ammonia availability in areas such as central valley, California leads to higher NOx BPTs. Note that benefits from emissions reductions in Canada are also seen when using U.S. health benefits due to long-range and cross-border transport of PM_2.5_ and its precursors.

Similar to the conclusions drawn for US-based BPTs, particulate matter and ammonia (Figure 2a,b) tend to have larger per tonne benefits than SO_2_ (Figure 2d) and NO_*x*_ (Figure 2c) when considering the Canadian population, though peak BPTs tend to be smaller, reflecting lower population and VSL estimates. The highest BPT values are seen over the urban centers (Vancouver, Ottawa, Montreal and Toronto), driven mainly by population density, since these four (4) cities account for a sizable portion of the total Canadian population. Since these urban centers are located very close to the border, benefits from emissions reductions extend into the U.S. states closest to Canada.

The results shown in Figures 1 and 2 are GEMM BPTs for surface sources. BPTs for elevated sources (see Appendix C) have similar patters to surface BPTs but decrease with altitude as expected; however, the rate of this reduction with altitude is gradual and varies for different emitted species. In other words, while surface and aloft BPTs are different, these differences are not substantial, particularly for shorter stack heights.

Primary emission BPTs are almost invariably positive, but BPTs of precursor emissions may be occasionally negative. For example, in NO_*x*_-rich environments or plumes, a negative impact of NO_*x*_ on ozone can also reduce nitrate formation, particularly at night, resulting in negative BPTs. Similarly, in ammonia-limited environments, a reduction in SO_2_ emissions may, in rare occasions, result in increased particle mass through nitrate formation, again resulting in a negative BPT.

Adjoint-based BPTs represent source impacts, and although BPTs are defined as benefits of emission reductions, they can also be viewed as the damage or burden associated with increased emissions. As potential source impacts, BPTs can exist at any location, regardless of the level of emissions at that location. Therefore, having sizable BPTs over the ocean or uninhabited areas is possible. However, depending on the lifetime of primary emissions, or conversion time to PM_2.5_ for precursor emissions, BPTs are more likely to follow, to varying extents, the spatial distribution of population. For example, BPTs for primary PM_2.5_ emissions often closely follows the population centers, as emissions are expected to result in the largest exposure in the vicinity of surface sources.

**Figure 3:**
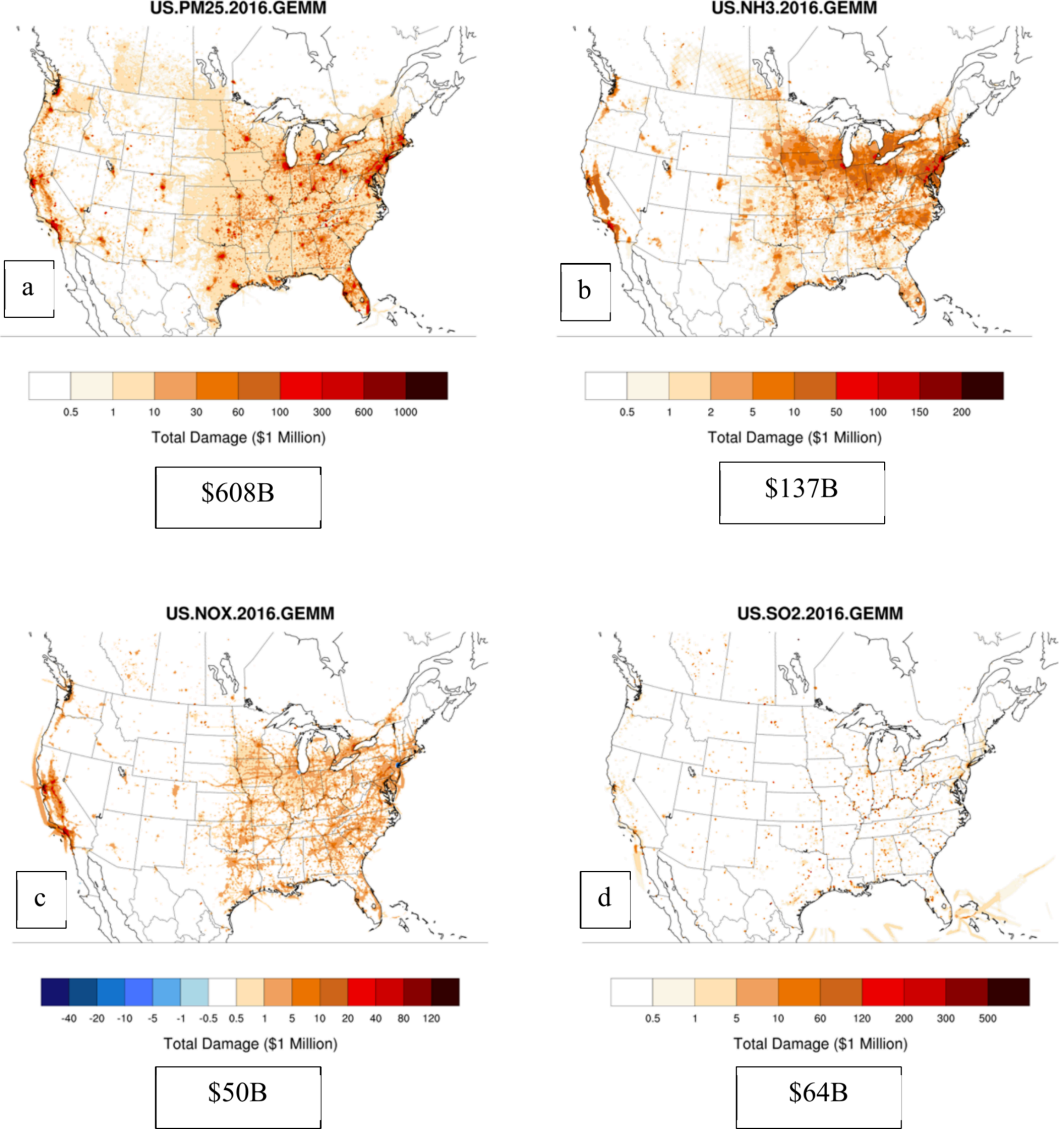
Spatial maps of first-order location-specific burden estimates for the U.S. for PM_2.5_ (a), NH_3_ (b), NO_*x*_ (c), and SO_2_ (d). Total, emissions-specific, damages across the domain are reported in 2016 USD in the text-box below each plot.

**Figure 4:**
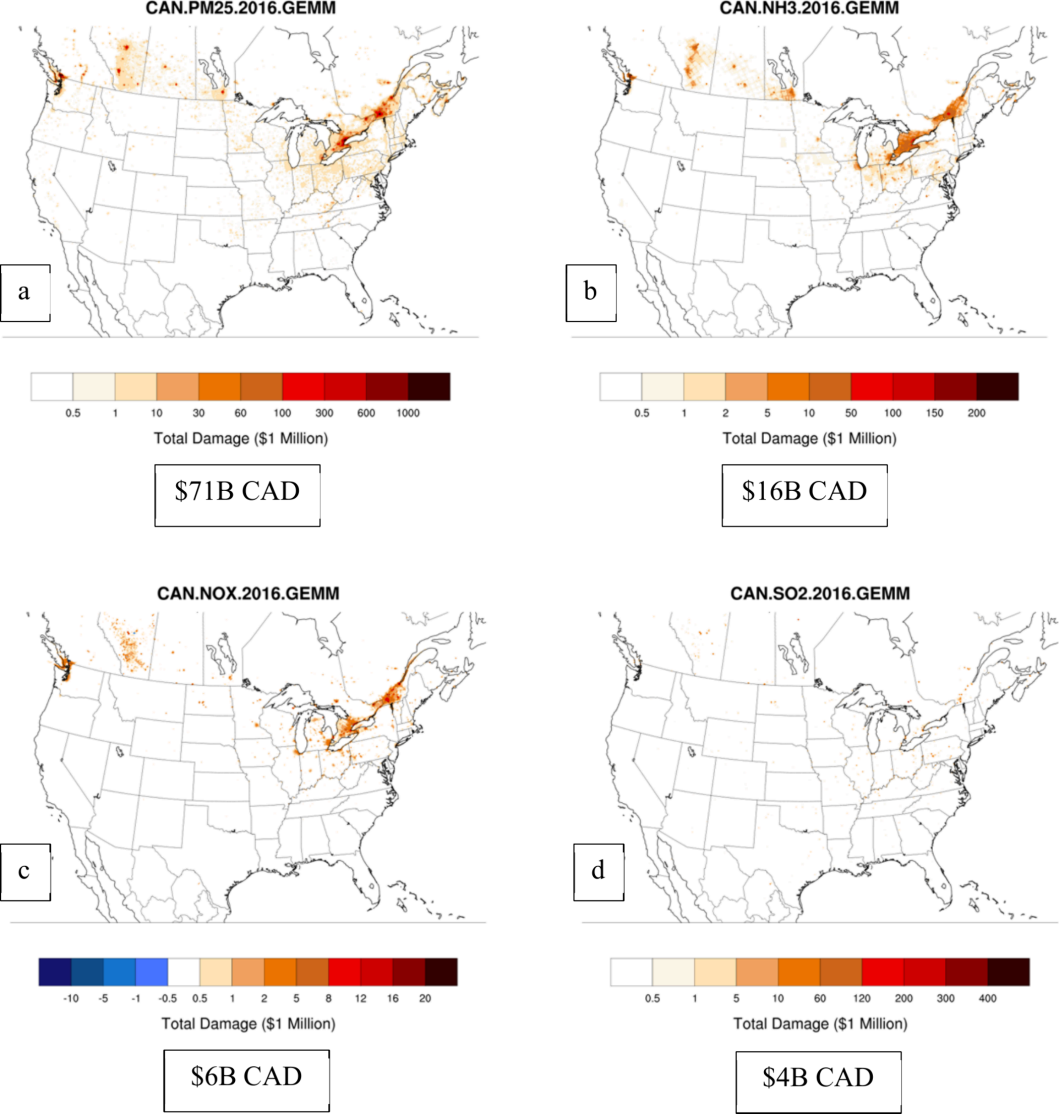
Spatial maps of first-order location-specific burden estimates for Canada for PM_2.5_ (a), NH_3_ (b), NO_*x*_ (c), and SO_2_ (d). Total damages across the domain are reported in 2016 CAD in the text-box below each plot.

For both Canada and the U.S., PM_2.5_ and ammonia have the largest BPTs. However, the total burden associated with the emissions of each species also depend on the magnitude of emissions. As measures of response at the margin, BPTs represent the slope or tangent to the model’s response surface (or the sensitivity). However, if for a nonlinear response surface, BPTs are used to characterize source impacts in the presence of large-scale changes in emissions, their use can lead to errors due to nonlinearity if very large changes in emissions are being examined. In the presence of significant nonlinearities, or large-scale changes in emissions, or both, the BPTs (or tangents to the surface) may deviate from changes between the two emission points. Nonlinearity in the health response to an emissions change can stem from nonlinear atmospheric processes (mainly gas-phase and aqueous chemistry and aerosol thermodynamics) or from a nonlinear CRF such as GEMM. While BPTs represent marginal source impacts, to a first-order approximation the overall burden from emissions of a species can be estimated using BPTs. The burden can be approximated as a product of BPT and emissions at any given location. Here, we use this first-order approximation, while recognizing its inherent limitations (Figures 3 and 4, total surface emissions burden estimates for Canada and the U.S.).

In a similar fashion to the BPTs from Figure 1, the spatially resolved total burden estimates are highest for locations with the highest emissions activity and population (California and the Eastern US) (Figure 3). Particulate matter emission reductions (Figure 3a) have the largest overall potential benefits, totaling more than $600B 2016 USD in health benefits over the entire U.S. under a zero-emissions case, followed by ammonia (Figure 3b) with 137 billion USD. Reductions in NO_*x*_ (Figure 3c) and SO_2_ (Figure 3d) emissions can also yield significant benefits, albeit smaller than the equivalent reductions in PM_2.5_ and ammonia.

For the U.S., we estimate a total burden of 91,000 premature mortality (including secondary organic PM_2.5_ from VOCs) from all natural and anthropogenic sources in the U.S., comparable to past studies.^44,45^ Total burden estimates for Canada (Figure 4) mirror those of the US; PM_2.5_ still dominates the other species (Figure 4a), followed by NH_3_ (Figure 4b), NO_*x*_ (Figure 4c), and SO_2_ (Figure 4d).

### 3.1 Emissions Are Not Created Equal

**Figure 5:**
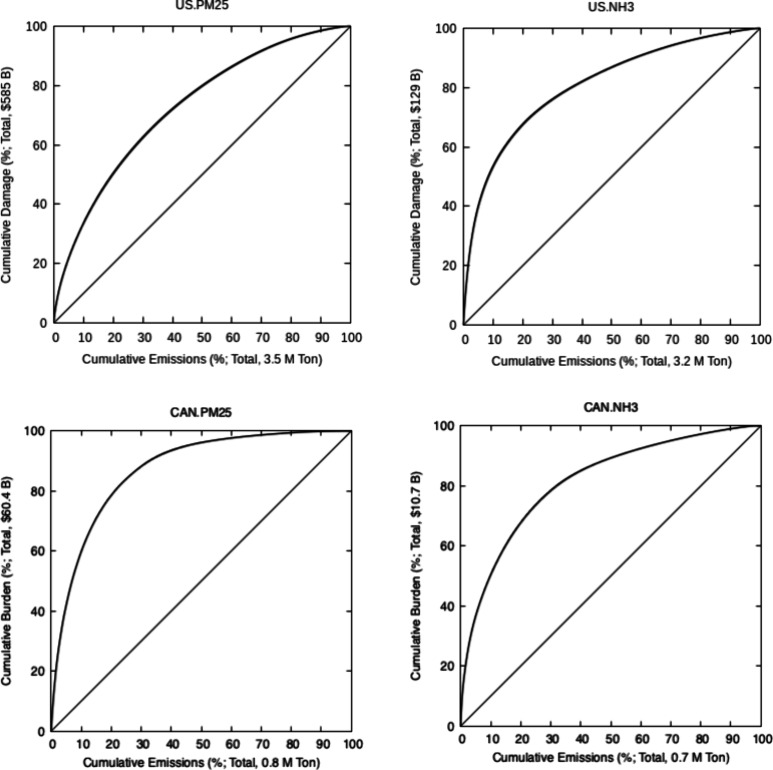
Lorenz curves for the U.S. and Canada display the extent of inequality in health burden of emissions across the two countries for U.S. PM_2.5_ (a) and NH_3_ (b) and Canadian PM_2.5_ (c) and NH_3_ (d) emissions. The vertical and horizontal axes show cumulative burden and cumulative emission fractions, respectively. Cross-border impacts are not included in these curves, and therefore, total burdens are smaller than those in Figures 3 and 4 which depict nationwide burdens from domain-wide emissions.

Location-specific BPTs provide granular differentiation between source impacts. The level of spatial detail can provide valuable information for guiding air quality management decisions. This added value is evident as BPTs in both Canada and the U.S. exhibit significant spatial and seasonal variability. Lorenz curves^46^ are developed by rank-ordering the locations from which emissions have the highest impacts (Figure 5). Lorenz curves are often used to display disparity in income or wealth in a population. In an analogous manner, Lorenz curves in Figure 5 can be viewed as disparity of health impacts among different tonnes of emissions in all locations in Canada or the U.S. Our results (Figure 5) suggest that in the U.S., 20% of primary PM_2.5_ (Figure 5a) and ammonia (Figure 5b) emissions are responsible for 50% and 60% of health burden of those species, respectively. Similarly in Canada, the most harmful 10% of PM_2.5_ (Figure 5c) and ammonia (Figure 5d) emissions account for approximately 60% and 50% of the total burden, respectively.

**Figure 6:**
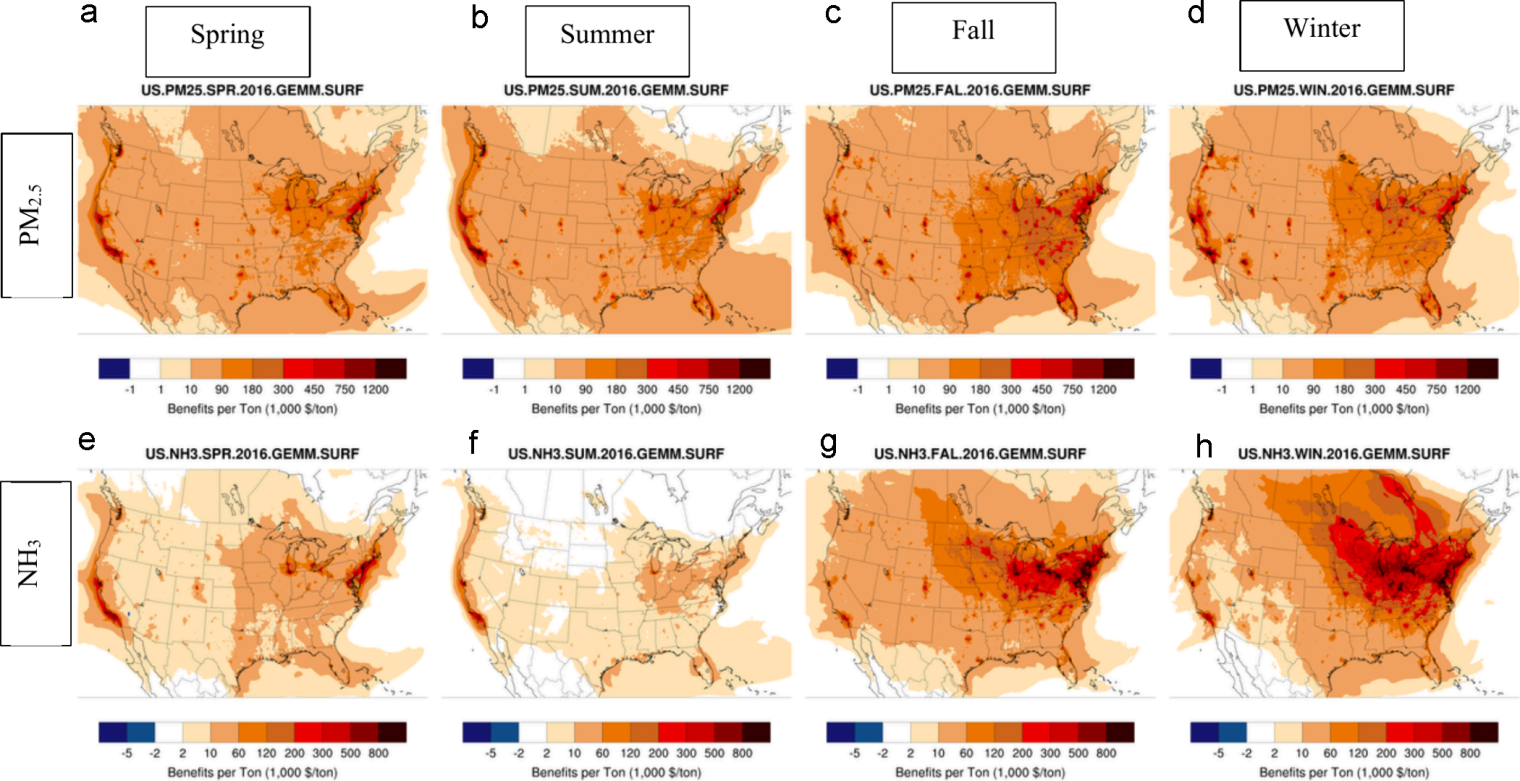
Variability in BPTs of primary PM_2.5_ and ammonia for the Spring (a and e), Summer (b and f), Fall (c and g), and Winter (d and h) seasons.

All the BPTs shown above have been averaged over four seasons to construct annual values. In addition to spatial variability, BPTs also display significant seasonal variability (Figure 6), especially for secondary inorganic aerosol precursors, whose partitioning is a function of temperature. Ammonia shows the most significant seasonal trends with larger BPTs during the colder winter and fall seasons (Figure 6e-h), where lower temperatures favor partitioning to particulate ammonium, and contributing to nitrate formation. Note that while ammonia BPTs are largest in cold seasons, emissions of ammonia are lowest in those conditions. Primary PM_2.5_ emissions, on the other hand, show little seasonality (Figure 6a-d) as their impact is affected not by the nonlinear chemical or thermodynamic transformations, but only through seasonal weather parameters and mixing patterns. Despite relative seasonal stability, some differences in seasonal BPTs for primary PM_2.5_ emissions can be seen in certain regions (e.g., increased BPTs in Midwest and Northeast in colder seasons) which may be due to change in seasonal meteorological conditions such as lower mixing height or altered wind patterns.

## 4 Discussion

The adjoint model provides a unique approach for the estimation of location-specific source impacts within a full-complexity modeling framework. Location-specific BPTs, as estimated by the adjoint model, show a great deal of spatial and temporal variability and can be used in guiding targeted emission control policies. We find that the largest potential benefits, per tonne of emission reduced, can be realized through reductions in primary particulate matter both for the U.S. and Canada (up to more than 1 million USD per ton), followed by reductions in ammonia (up to 800,000 USD per ton). Reduced SO_2_ and NO_*x*_ emissions also yield benefits, although at a value per ton smaller than the ones from PM_2.5_ and ammonia. As expected, BPTs tend to have the largest magnitude in locations with the most proximity to the largest population, as evident by the total amount of monetized benefits that can be reaped from potential reductions. Due to the inextricable nature of emissions/population, not all emissions of a species have equal health impacts, and therefore the greatest benefits can be achieved by reducing the most harmful portion of emissions, rather than indiscriminate control of emissions. For example in Canada, a 10% reduction in most damaging primary PM emissions can negate approximately 60% of the total health burden. Seasonality may also be important with regards to BPTs, especially as it pertains to secondary aerosol species, since the lower temperatures during the winter and fall promote partitioning and formation of secondary species.

BPT values have often been estimated for emissions of various sectors.^3,4,47^ However, at any given time and location (including altitude), source impacts for an emitted pollutant are independent of the sector and type of the source as the atmosphere does not distinguish between the origin of the pollutant. What leads to sectoral differences are the different spatial and temporal emission patterns for sources that are grouped together. One of the advantages of developing adjoint-based BPTs is that they are specific to locations and emission species (e.g., NOx, NH_3_, PM_2.5_ direct emissions). As a result, differences in annually averaged adjoint-based BPTs at each location can only exist due to sector-specific temporal patterns. Since temporal fingerprints of most source sectors are far less pronounced than spatial features and patterns, adjoint BPTs at a specific location are generally applicable to various source sectors (see SI, Appendix D for an example). For sectors that have very distinct seasonal and/or temporal pattern of emissions (e.g., school buses or residential wood combustion), more accurate BPTs can be estimated from hourly or seasonal BPTs as weighted averages based on emissions of those sectors (see Supporting Information and Data Repository).

We note that our burden estimates for the U.S. and Canada (Figures 3 and 4) do not include VOC burden, nor do we report VOC BPTs. The version of CMAQ used in the adjoint model does not include a range of updates for oxidation pathways leading to anthropogenic and biogenic secondary organic aerosol (SOA) formation,^48−52^ and as such we refrain from reporting VOC BPTs here. Furthermore, VOC composition varies greatly across sectors, and because various VOC species can have vastly different BPTs, reporting a single set of location-specific VOC BPTs that is applicable to multiple sectors is not possible.

The version of the adjoint model used is also likely to have an impact on the estimated burden of primary PM_2.5_ emissions, as later versions of CMAQ (v5.2 and later) account for evaporation of primary organic emissions into semivolatile organic compounds and their subsequent oxidation to SOA.^52^ As a result, some of the burden attributable to SOA in later versions of CMAQ are assigned to primary PM_2.5_ emissions in our results. We note that this issue would only impact the attribution of burden and is not likely to affect the estimated BPTs for primary PM_2.5_ emissions.

Location-specific BPTs offer obvious value in informing decisions that target more impactful emissions. A potentially important application for location-specific BPTs is the estimation of location- and sector-specific cobenefits stemming from decarbonization and CO_2_ reductions. Such location-specific cobenefits can be regarded as a form of BPTs (per tonne of CO_2_) that aggregate the health impact over all species. Unlike BPTs, location-specific cobenefits have strong sectoral footprints and are substantial for sectors such as off-road engines and diesel heavy duty vehicles, and in most urban locations they can far exceed estimates of the social cost of carbon. Within the decarbonization context, these cobenefits can offer invaluable potential for harvesting significant population health benefits from GHG mitigation policies.

Our results are subject to a range of uncertainties stemming from inputs, models, assumptions, and natural variability. Similar to any CTM-based study, uncertainties and errors in emissions inventories, emissions modeling, meteorological modeling, atmospheric process representation and formulation, numerical solutions, etc., contribute to uncertainties in estimated concentrations and exposures. Using VSL for monetizing health impacts introduces additional uncertainties in estimated BPTs. Our adjoint simulations are performed at the highest feasible resolution, but the resolution is still less than ideal for adequate population exposure assessment. Epidemiological uncertainties, both in magnitude of effect estimates and the shape of CRF, are likely to be significant contributors to the overall uncertainty. Our estimates of BPTs are for a single year, and their use for other years would introduce additional uncertainties due to interannual variability or changes in atmospheric state. Finally, the treatment of SOAs in the version of the adjoint model used lags behind newer versions of CMAQ, and can benefit from updates to the code. Quantifying the impact of these uncertainties is a challenging undertaking. The impact of critical assumptions (episodic simulations, resolution, choice of CRF, inventory level) on estimated BPTs is examined in a separate work.^21^

## Data Availability

BPT estimates (surface and aloft) for GEMM and other CRFs for Canada and the U.S. are available at 10.5683/SP3/DTS44O.

## Supporting Information Available

Additional details about episode selection; BPTs of extra CRFs; BPTs for elevated emission sources (PDF)
