# Supplementary material for: Spatiotemporally Detailed Quantification of Air Quality Benefits of Emissions Reductions–Part I: Benefit-per-Ton Estimates for Canada and the U.S
Source: ACS EST Air. 2024 Sep 3;1(10):1215–26. doi: 10.1021/acsestair.4c00127 (PMC11474827; doi:10.1021/acsestair.4c00127)
Supplement: Supplementary file 1 — ea4c00127_si_001.pdf [file ea4c00127_si_001.pdf]

Supplementary Information

for

Spatiotemporally Detailed Quantification of Air Quality Benefits of Emissions Reductions – Part I: Benefit-per-Ton Estimates for Canada and the U.S.

Shunliu Zhao[a], Petros Vasilakos[b#], Anas Alhusban[a], Yasar Burak Oztaner[a], Alan Krupnick[c], Howard Chang[d], Armistead Russell[b], Amir Hakami[a]*

[a]Department of Civil and Environmental Engineering, Carleton University, Ottawa, ON K1S 5B6, Canada

[b] School of Civil and Environmental Engineering, Georgia Institute of Technology, Atlanta, GA 30331, USA

[c] Resources For the Future, Washington , DC 20036, U.S.A.

[d] Emory University, Atlanta, GA 30322, U.S.A.

Appendix A: Episode selection

For the anomaly analysis for episode selection, we use two normalized bias functions, one for domain-wide bias in seasonal BPTs and one for bias in burden, as defined as the product of seasonal BPT and emissions for each grid cell in the model:

$$f_{BPT,t} = \frac{100}{N_{grids} \times N_{spc}} \sum_{spc} \sum_{grids} \frac{(BPT_{grid,spc,t} - BPT_{grid,spc,season})}{BPT_{grid,spc,season}} \quad (1)$$

and,

$$f_{burden,t} = \frac{100}{N_{grids} N_{spc}} \sum_{grids} \frac{(\sum_{spc} BPT_{grid,spc,t} E_{grid,spc,t} - \sum_{spc} BPT_{grid,spc,season} E_{grid,spc,season})}{\sum_{spc} BPT_{grid,season} \times E_{grid,season}} \quad (2)$$

where $BPT_{grid,spc,t}$ is the BPT estimate for a specific grid cell and emitted species and for the episode starting on day t of the season, while $BPT_{grid,season}$ is the same estimate when all days of the season are included in the estimate, and E_{grid} values are the daily or seasonal average emission rates of an emitted species. Normalized bias functions $f_{BPT,t}$ and $f_{burden,t}$ are calculated for each day and are expressed as percentages. Primary $PM_{2.5}$, SO_2, NO_x, NH_3 are the species that are included in constructing bias functions. Only surface BPT and burden estimates are used in constructing bias functions.

The episode that minimizes the summation of these two bias functions is then chosen as the representative episode for the season:

$$min. (\frac{f_{BPT,t} - f_{BPT,t,min}}{f_{BPT,t,min}} + \frac{f_{burden,t} - f_{burden,t,min}}{f_{burden,t,min}}). \quad (3)$$

where $f_{BPT,t,min}$ and $f_{burden,t,min}$ are the minimum daily BPT and burden biases for the season. These bias functions were chosen for spatiotemporal anomaly analysis based on trial and error of various measures of deviation, as well as different weights for the two bias functions. While our choice or bias function weighting is arbitrary, in our trail and error we found that selected episodes are not overly sensitive to that choice. Note that our anomaly analysis is based on 36 km BPTs, and therefore, our episodic representation of seasonals BPTs entails three assumptions that a) the chosen episode is representative of the season, and b) temporal patterns of 12 km BPTs are consistent with those of 36 km estimates, and that selected episodes can be applied nationally without significant loss of regional representativeness. We conduct sensitivity analysis to evaluate errors associated with these assumptions and episodic representation of seasons.

The episodes are selected for Canada and the U.S. independently. Based on bias functions derived above (Figure A1) the episodes for the two countries are shown in Table A1:

Table A1: Selected episodes for Canada and the U.S.

Episode selection		
	USA	Canada
Winter	02/12/2016 - 02/25/2016	01/30/2016 - 02/12/2016
Spring	05/04/2016 - 05/17/2016	05/04/2016 - 05/17/2016
Summer	08/02/2016 - 08/15/2016	08/03/2016 - 08/16/2016
Fall	11/16/2016 - 11/29/2016	11/18/2016 - 12/01/2016

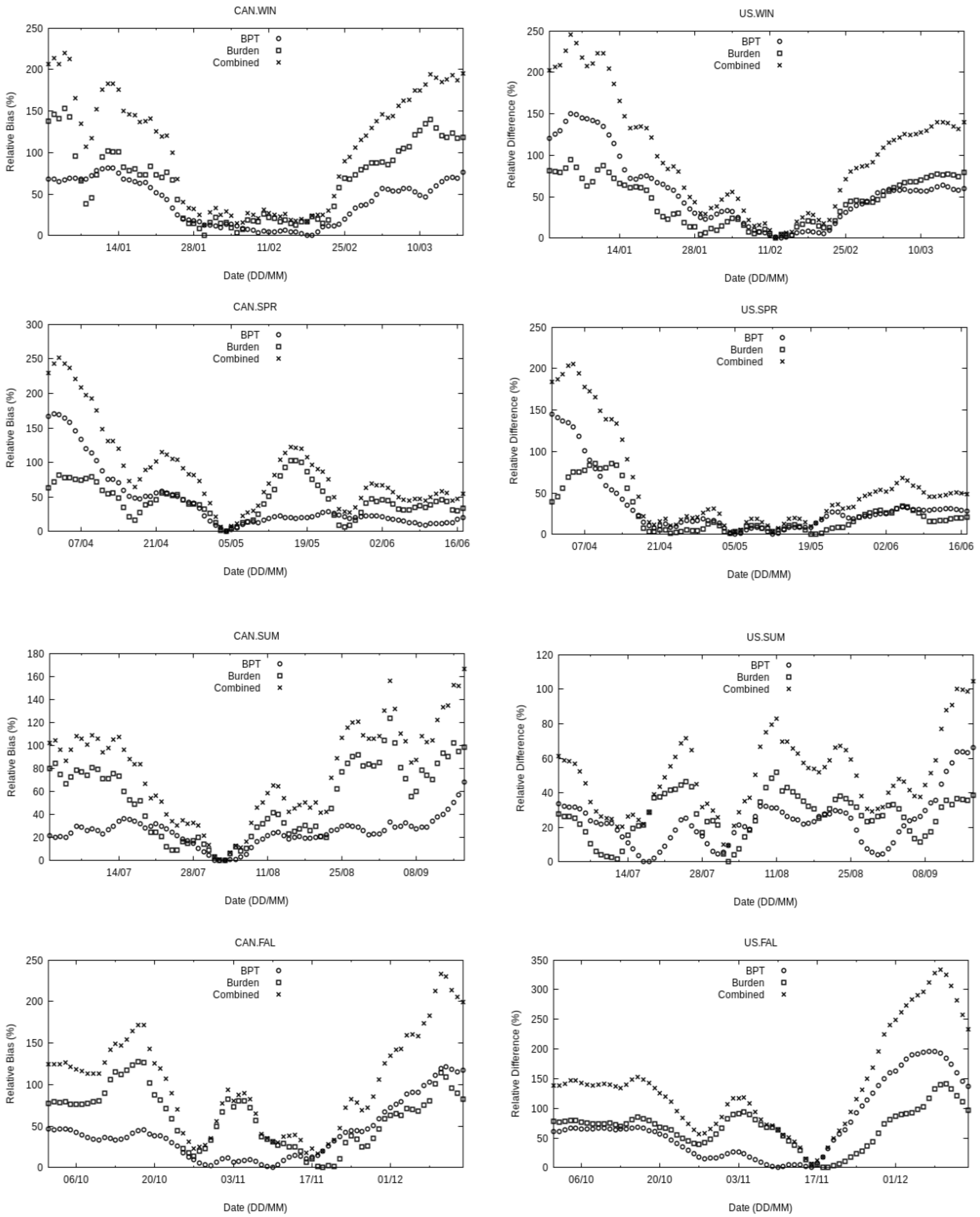

Figure A1: Summary of seasonal bias functions for episode selection in Canada and the U.S. Each point on the plot indicates normalized bias (BPT, burden, or combined) for a two-week period starting with the date.

Appendix B: BPTs of CRFs other than GEMM

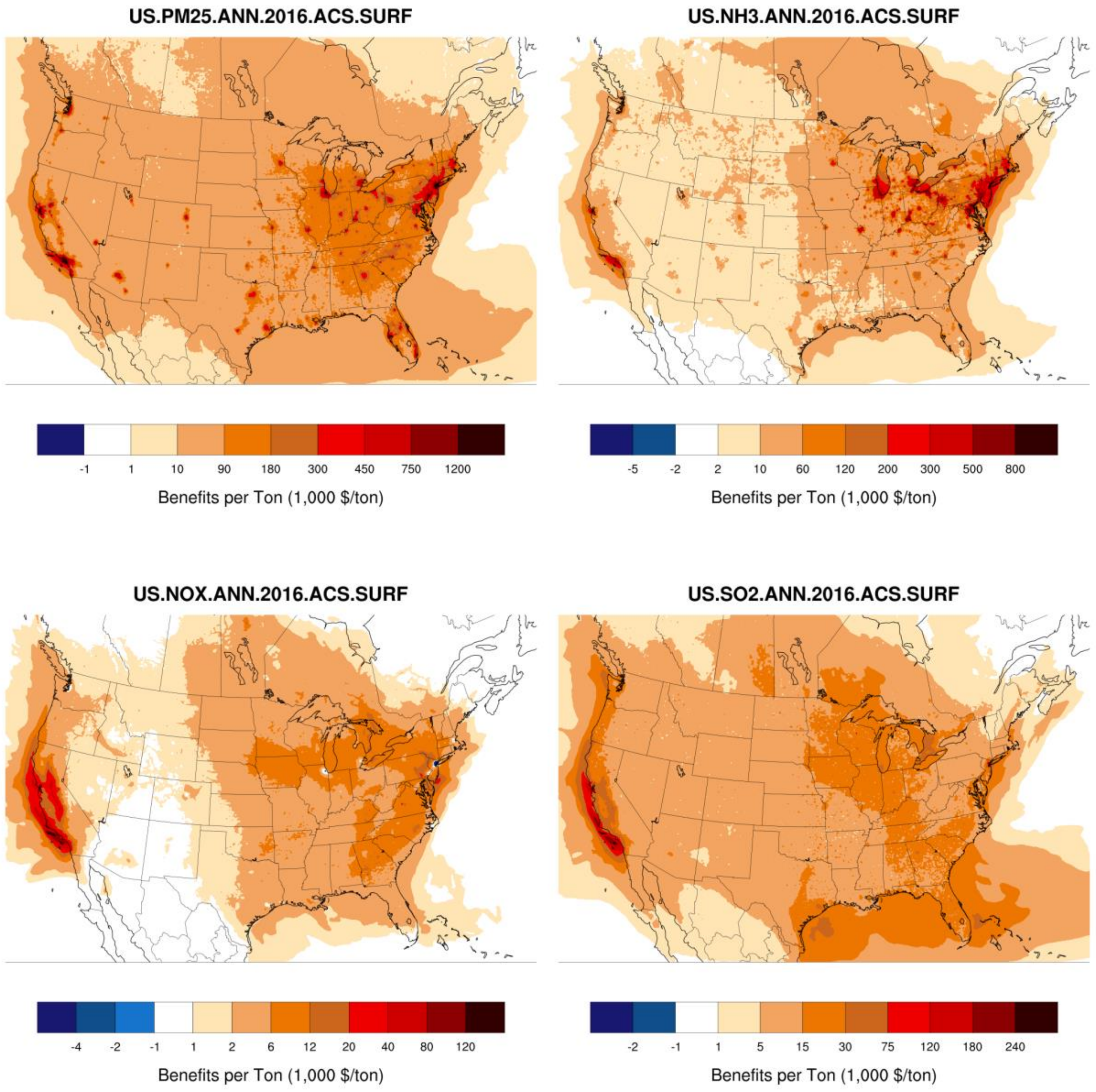

Figure B1: Annual BPT estimates, U.S., surface, ACS-09 CRF.[1]

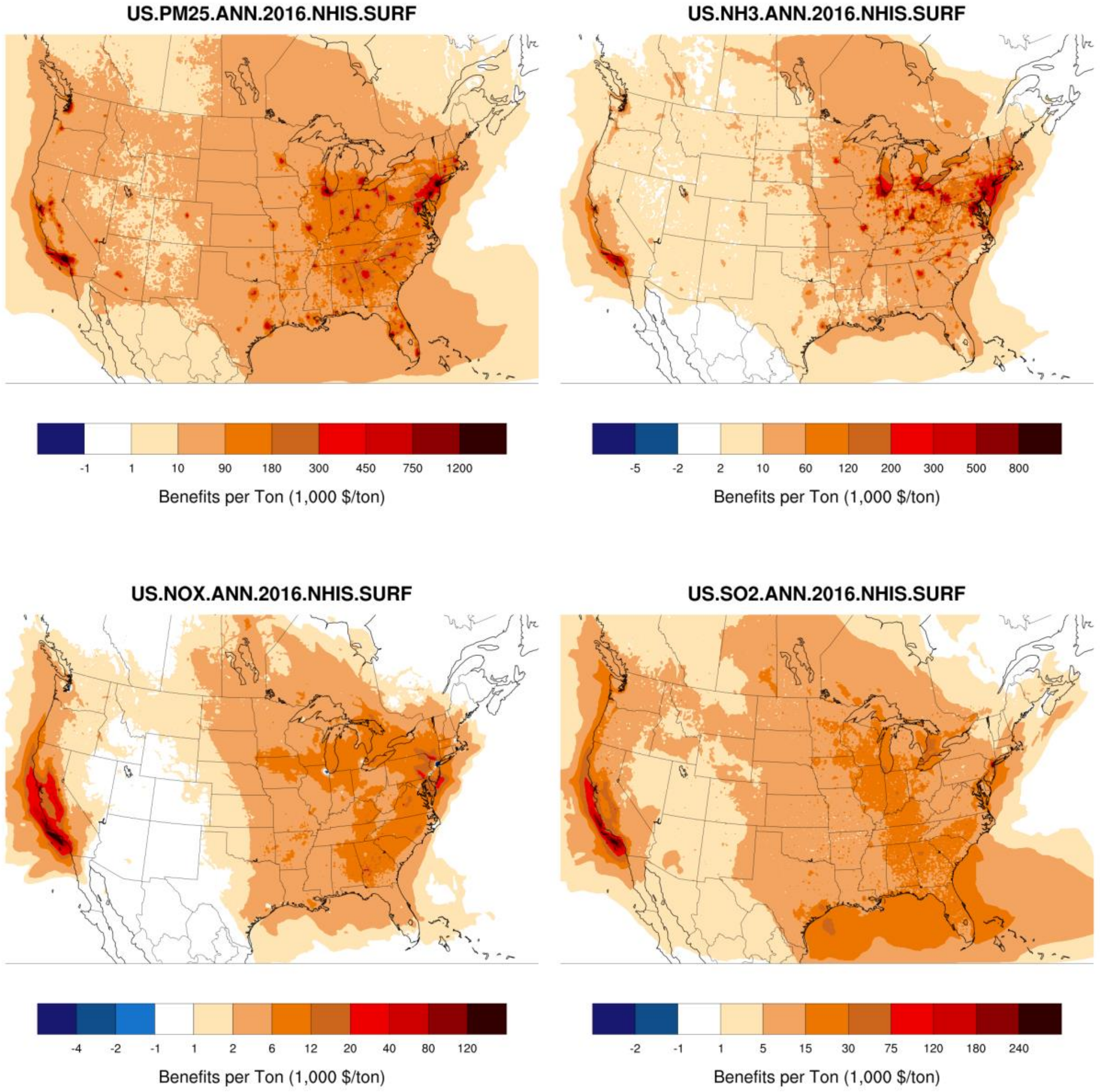

Figure B2: Annual BPT estimates, U.S., surface, NHIS CRF.[2]

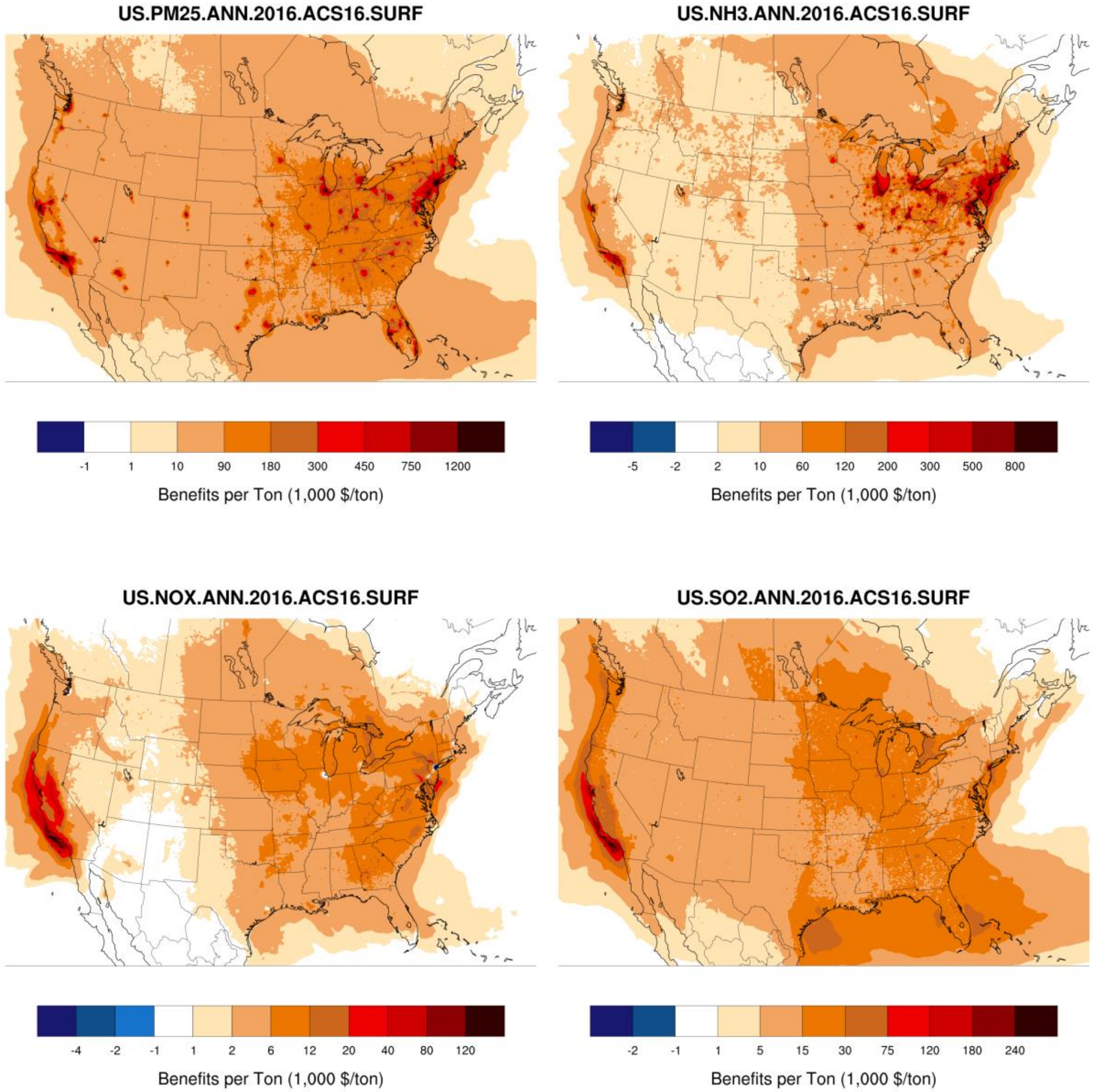

[2]

Figure B3: Annual BPT estimates, U.S., surface, ACS-16 CRF.[3]

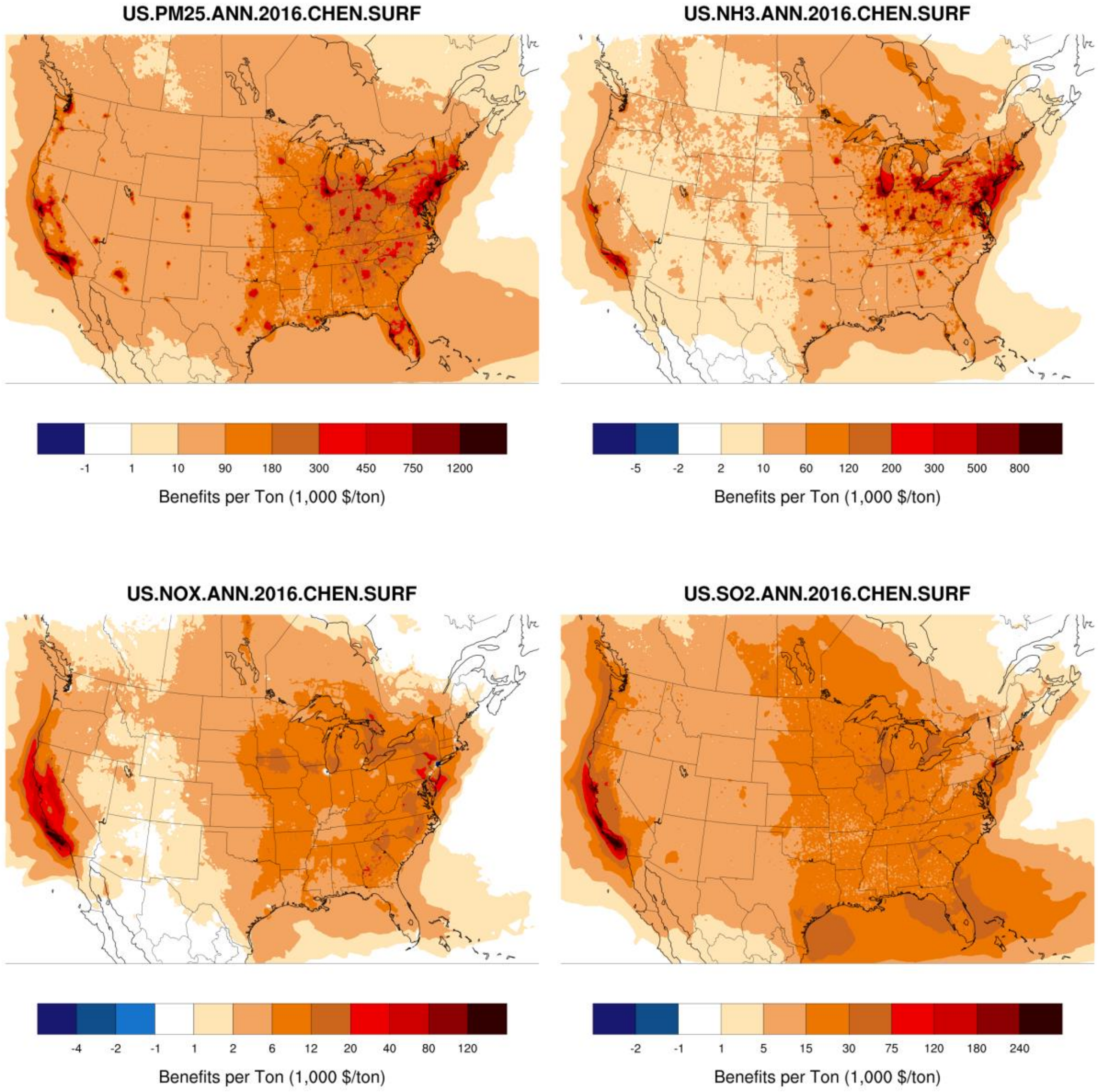

Figure B4: Annual BPT estimates, U.S., surface, CHEN CRF.[4]

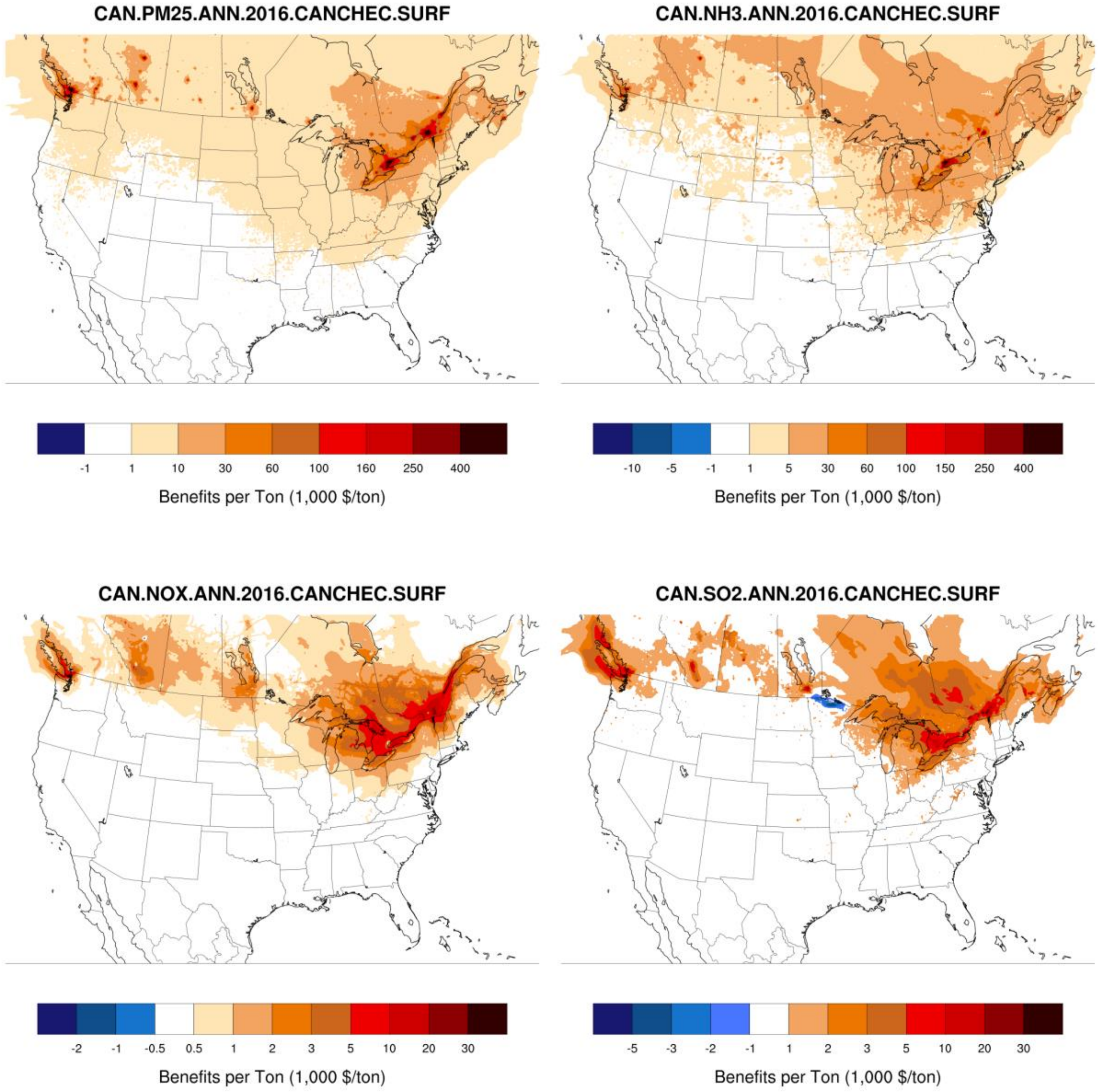

Figure B5: Annual BPT estimates, Canada, surface, CanCHEC CRF.[5]

References:

(1) Krewski, D.; Jerrett, M.; Burnett, R. T.; Ma, R.; Hughes, E.; Shi, Y.; Turner, M. C.; Pope III, C. A.; Thurston, G.; Calle, E. E. *Extended Follow-up and Spatial Analysis of the American Cancer Society Study Linking Particulate Air Pollution and Mortality*; Health Effects Institute Boston, MA, 2009; Vol. 140.

(2) Pope, C. A.; Lefler, J. S.; Ezzati, M.; Higbee, J. D.; Marshall, J. D.; Kim, S.-Y.; Bechle, M.; Gilliat, K. S.; Vernon, S. E.; Robinson, A. L.; Burnett, R. T. Mortality Risk and Fine Particulate Air Pollution in a Large, Representative Cohort of U.S. Adults. *Environmental Health Perspectives* **2019**, *127* (7), 077007. https://doi.org/10.1289/EHP4438.

(3) Turner, M. C.; Jerrett, M.; Pope, C. A.; Krewski, D.; Gapstur, S. M.; Diver, W. R.; Beckerman, B. S.; Marshall, J. D.; Su, J.; Crouse, D. L.; Burnett, R. T. Long-Term Ozone Exposure and Mortality in a Large Prospective Study. *Am J Respir Crit Care Med* **2016**, *193* (10), 1134–1142. https://doi.org/10.1164/rccm.201508-1633OC.

(4) Chen, J.; Hoek, G. Long-Term Exposure to PM and All-Cause and Cause-Specific Mortality: A Systematic Review and Meta-Analysis. *Environ Int* **2020**, *143*, 105974. https://doi.org/10.1016/j.envint.2020.105974.

(5) Crouse, D. L.; Peters, P. A.; van, D. A.; Goldberg, M. S.; Villeneuve, P. J.; Brion, O.; Khan, S.; Atari, D. O.; Jerrett, M.; Pope, C. A.; Brauer, M.; Brook, J. R.; Martin, R. V.; Stieb, D.; Burnett, R. T. Risk of Nonaccidental and Cardiovascular Mortality in Relation to Long-Term Exposure to Low Concentrations of Fine Particulate Matter: A Canadian National-Level Cohort Study. *Environmental Health Perspectives* **2012**, *120* (5), 708–714. https://doi.org/10.1289/ehp.1104049.

Appendix C: BPTs of elevated sources

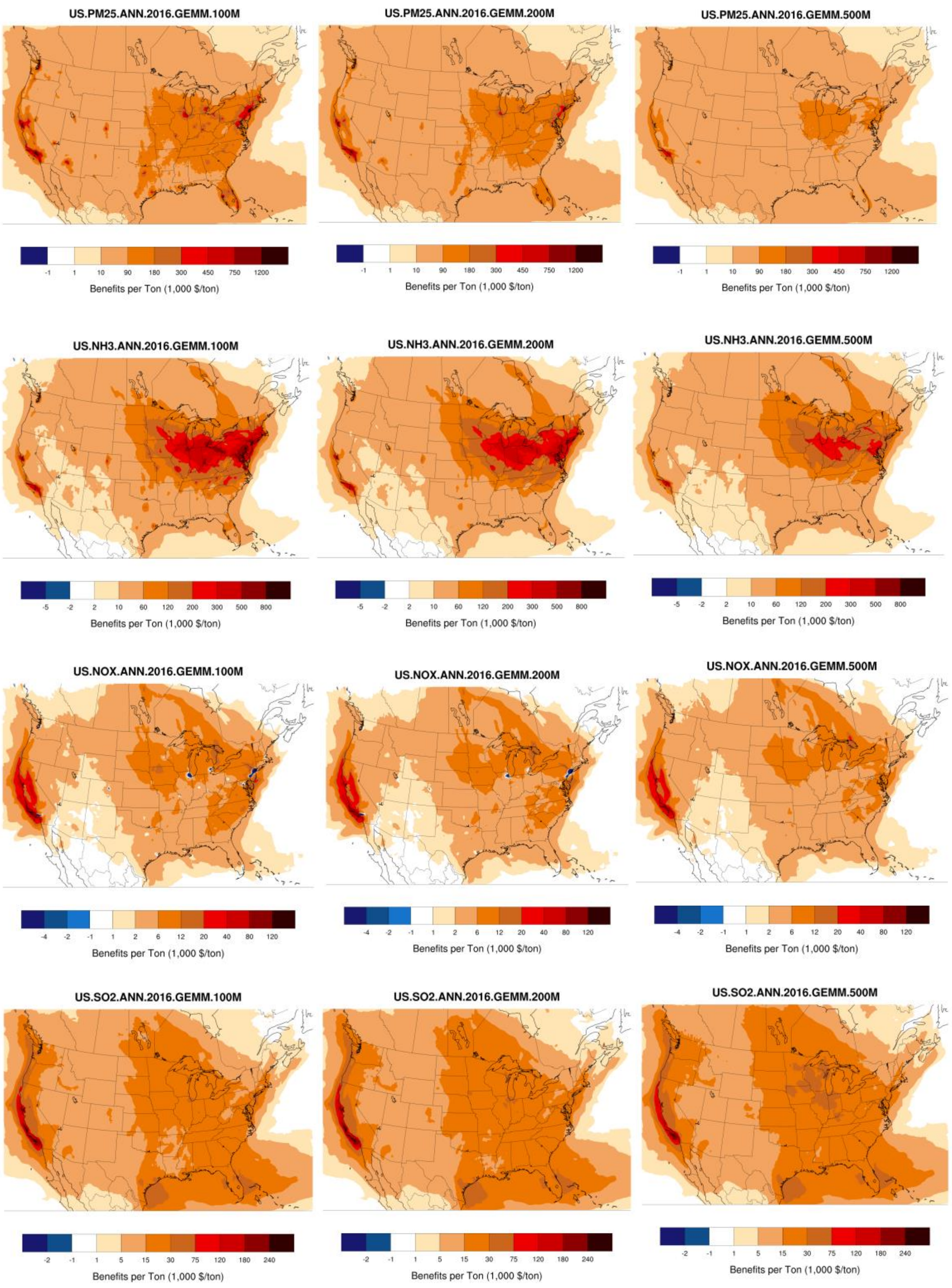

Figure C1: BPTs for elevated sources at 100m, 200m, and 500m elevation, U.S., GEMM CRF.

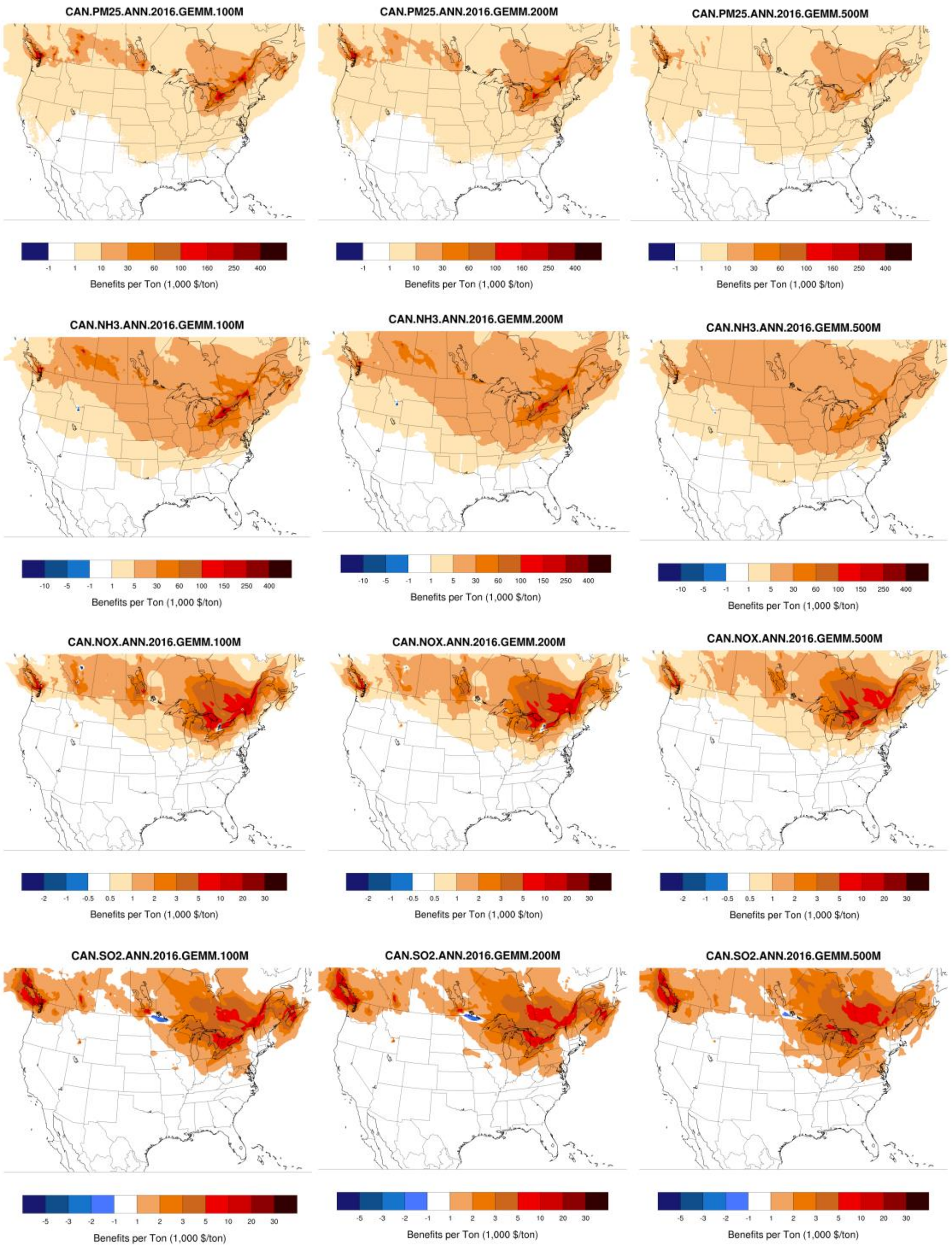

Figure C2: BPTs for elevated sources at 100m, 200m, and 500m elevation, Canada, GEMM CRF.

Appendix D: Example of sectoral BPTs for select internal combustion engines

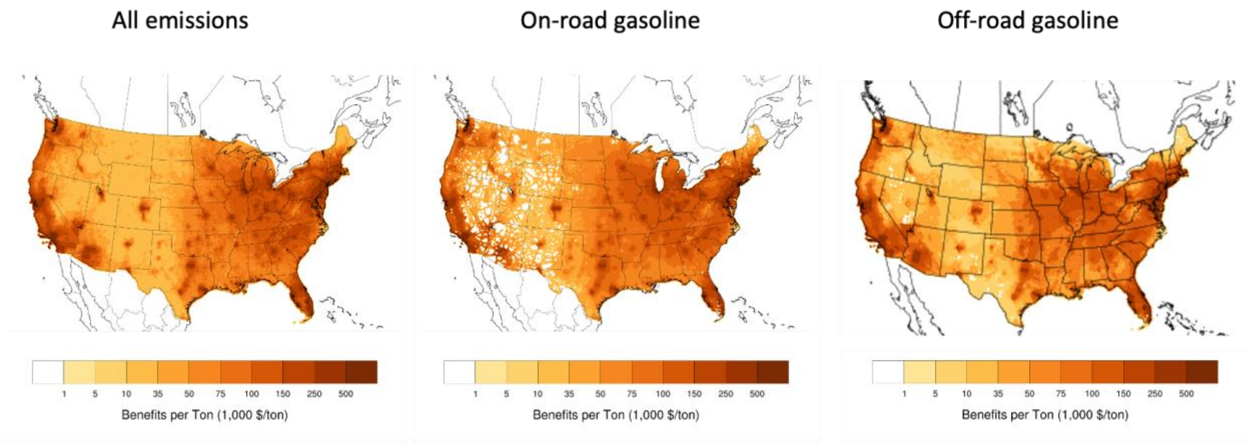

Figure D1: Example of differences in sectoral BPTs for on-road and off-road gasoline vehicles, and those calculated from all mobile emissions. For location-specific BPTs, differences in sectoral BPTs only arise from differences in temporal patterns, which are rather minor in nature.
